# A sensory assessment of color and textural quality of refrigerated tomatoes preserved with different concentrations of potassium permanganate

**DOI:** 10.1002/fsn3.410

**Published:** 2016-08-01

**Authors:** Victor C. Wabali, Akpevwe Esiri, Leelee Zitte

**Affiliations:** ^1^ Department of Crop/Soil ScienceFaculty of AgricultureUniversity of Port‐HarcourtPort‐HarcourtNigeria; ^2^ Department of Animal and Environmental BiologyFaculty of Biological SciencesCollege of Natural and Applied SciencesUniversity of Port‐HarcourtPort‐HarcourtNigeria

**Keywords:** Postharvest preservation, tomatoes, shelf life and quality

## Abstract

Freshly harvested mature tomato fruits were treated with different concentrations of potassium permanganate to evaluate their effect on color and texture of the fruits. This was to determine the degree of acceptability and shelf life of the tomatoes. Fifty grams of mature unripe tomato fruits was washed and weighed into a transparent plastic container containing different concentrations of potassium permanganate (control, 2.5 ppm, 5.0 ppm, 7.5 ppm. 10.0 ppm, 12.5 ppm, and 15.0 ppm). The experiment was carried out in a complete randomized design and replicated four times. All the treatments were refrigerated at a temperature range 14–18°C and a sensory assessment of color and textural changes was carried out through a team of selected panelists using the hedonic scale ranking to determine the degree of acceptability. Results obtained were analyzed using analysis of variance DMRT at 5% level of probability and least significant difference (LSD). Results obtained indicated that 7.5 ppm of potassium permanganate had a preservative effect on color and texture of refrigerated tomatoes. Therefore, the tomatoes were of acceptable color and texture for a period of 21 days as revealed in the result. Color quality had a mean score of 1.64 and texture ranking was 1.73 after 3 weeks of storage.

## 1 Introduction

Nutritionally, tomatoes constitute a source of vitamins and minerals in the human diet and have become one of the major vegetable crops cultivated. However, most of the tomatoes cultivated are lost as a result of poor preservative measures especially in developing countries like Nigeria. Poor infrastructure, inadequate preservative method, and poor handling processes during transportation and distribution have contributed substantially to food losses in Nigeria. For some perishable produce, about 40% of the harvested produce is lost due to spoilage after harvest. Therefore, there is an urgent need to have tomatoes in a form that is fresh, wholesome, and acceptable in order to meet consumer needs.

The quality and nutritional value of fresh produce like Tomatoes is affected by postharvest handling and storage conditions (Sablaini, Opara, & Al‐Balushi, 2006). In Africa one of the major contributing factors to food shortage and hunger is as a result of postharvest losses (Ojo, 1991). Consequently, developing methods, processes, and measures that will extend the shelf life of highly perishable crops like tomatoes have become imperative if food security will be achieved in Africa.

Postharvest loss of tomatoes in Nigeria is estimated to be about 40% annually (Okunoya, 1996). This occurs as a result of poor preservative methods and inadequate postharvest handling operations. Quality perception of tomatoes is viewed in terms of color and texture of tomatoes. Tomatoes deterioration is as a result of biochemical changes which affect color and textural properties. These changes alter cell‐wall polysaccharide composition, resulting in breakdown of cell‐wall polymers, such as cellulose, hemicelluloses, and pectin. (Kader, 1992; Khan, Prithiviray, & Smith, 2003). The changes play an important role in the shelf life of tomatoes. Fruits with different ripening mechanisms can be divided into two groups; climacteric, in which ripening is accompanied by a peak in respiration and concomitant burst of ethylene; and nonclimacteric in which respiration shows no dramatic change and ethylene production remains at a very low level (Alexander & Grierson, 2002). Ethylene is a plant hormone controlling a wide range of physiological processes in plants. During postharvest storage of fruits and vegetables ethylene can induce effects such as senescence, over ripening, and accumulated quality loss (Martinez‐Romero, Bailen, Serrano, Guillen, & Valverde, 2007).

Silva, Salomao, Dalmo, Paulo, and Rocha (2009) reported that potassium permanganate was effective in maintaining pawpaw at a preclimacteric stage for a 25‐day period, thereby extending the shelf life, and normal ripening was not interfered with after removal of the bags. Potassium permanganate oxidizes the ethylene produced by the fruit during ripening.

This study will evaluate the effectiveness of potassium permanganate in extending the shelf life of tomatoes in a form that is wholesome and acceptable to the consumers in terms of color and texture perception.

## 2 Materials and Methods

The materials used were mature unripe tomatoes, aerated pounches, plastic transparent containers, methylated spirit, cotton wool, and mettle top weighing balance. Potassium permanganate solution of different concentrations, masking tapes, and refrigerator. Different concentrations of potassium permanganate solutions were prepared (control, 2.5 ppm, 5.0 ppm, 7.5 ppm, 10.0 ppm, 12.5 ppm, and 15.0 ppm) and soaked in cotton wool. The experiment was laid out in a Complete Randomised Designed, with four replicates put inside aerated pounches and transferred into an already washed plastic transparent containers rinsed with methylated spirit. Fifty grams of mature unripe tomato fruits were washed, rinsed, and weighed with mettle top weighing balance and transferred to each of the plastic transparent containers containing different concentrations of potassium permanganate solution in aerated pounches and refrigerated at a temperature of 14–18°C. A 10 man selected team of panelists carried out sensory evaluation of the tomato fruits to determine changes in color and texture with a view to evaluate consumer acceptability for a period of 21 days.

A hedonic scale ranking was used to determine the level of changes in color (ripeness) or texture. Data collected daily for color on a hedonic scale ranking system of 1–5.

1 – Fresh

2 – Good

3 – Half ripe

4 – Ripe

5 – Bad

Textural quality was also evaluated, through sensory parameters by using a hedonic scale ranking of 1–6, where

1 – Hard

2 – Fairly hard

3 – Medium

4 – Fairly Soft

5 – Soft

6 – Bad

The data collected was analyzed using analysis of variance (DMRT) at 5% level of probability and Least Significant Difference (LSD).

## 3 Results

The results in Table 1 indicate that 7.5 ppm treatment performed better than the other samples for color acceptability, 7.5 ppm of KMnO_4_ showed no color change from 0 to 7 days, and after 8–11 days there was a change in color though not significant. Also color values for the tomatoes remained constant between 19 and 21 days.

**Table 1:** Effect of KMnO_4_ on the color acceptability of tomatoes under cold storage conditions

Days	Control	2.5 (ppm)	5 (ppm)	7.5 (ppm)	10 (ppm)	12.5 (ppm)	15 (ppm)
0–4	1.00	1.00	1.00	1.00	1.00	1.00	1.00
5	1.25	1.25	1.00	1.00	1.00	1.50	1.75
6	2.00	2.00	1.75	1.00	1.50	2.00	2.50
7	2.00	2.00	1.75	1.00	1.75	2.25	2.75
8	2.50	2.25	2.00	1.25	1.75	2.25	3.00
9	2.50	2.25	2.00	1.25	1.75	2.25	3.25
10	2.50	2.25	2.00	1.25	1.75	2.25	3.25
11	2.75	2.25	2.00	1.25	2.50	2.50	3.50
12	3.00	2.50	2.00	1.75	2.75	2.75	3.50
13	3.25	2.50	2.25	2.00	3.00	2.75	3.50
14	3.25	2.75	2.25	2.00	3.00	2.75	3.75
15	3.25	2.75	2.50	2.25	3.00	2.75	3.75
16	3.50	3.00	3.25	2.50	3.00	2.75	3.75
17	3.50	3.50	3.25	2.50	3.00	3.00	3.75
18	3.75	3.50	3.25	2.75	3.00	3.00	3.75
19	3.75	3.75	3.75	3.25	3.25	3.00	4.00
20	3.75	4.25	4.00	3.25	3.50	3.00	4.00
21	3.75	4.25	4.00	3.25	3.50	3.50	4.00
Mean	2.44	2.29	2.10	1.64	2.10	2.15	2.80

All textural scores were of acceptable standard after 21‐day period as indicated by Table 2. The treatment with 7.5 ppm concentration of KMNO_4_ had the best result when compared with other treatments as the fruits were harder. Textural quality remained the same as freshly harvested tomatoes between days 1 and 8, but did not differ significantly between days 8 and 11 for 7.5 ppm concentration.

**Table 2:** Effect of KMnO_4_ on the texture acceptability of tomatoes under cold storage conditions

Days	Control	2.5 (ppm)	5 (ppm)	7.5 (ppm)	10 (ppm)	12.5 (ppm)	15 (ppm)
0–3	1.00	1.00	1.00	1.00	1.00	1.00	1.00
4	1.50	1.25	1.00	1.00	1.25	1.50	1.75
5	1.50	1.25	1.00	1.00	1.25	1.50	1.75
6	2.00	1.75	1.75	1.00	1.25	2.00	2.50
7	2.00	1.75	2.00	1.00	1.25	2.25	3.00
8	2.25	1.75	2.00	1.00	1.25	2.25	3.00
9	2.75	2.25	2.00	1.25	1.75	2.25	3.25
10	2.75	2.25	2.00	1.25	1.75	2.25	3.50
11	2.75	2.25	2.00	1.25	2.25	2.50	3.50
12	3.00	2.75	2.00	1.50	2.25	2.50	3.75
13	3.00	3.00	2.00	1.75	2.50	2.75	3.75
14	3.25	3.25	2.50	2.00	2.75	2.75	4.00
15	3.50	3.25	2.50	2.25	3.00	2.75	4.00
16	3.75	3.50	3.00	2.50	3.00	2.75	4.00
17	4.00	3.50	3.00	2.50	3.00	2.75	4.25
18	4.25	3.50	3.00	2.75	3.00	2.75	4.25
19	4.25	4.00	3.75	3.50	3.25	2.75	4.50
20	4.25	4.00	3.75	3.75	3.25	3.50	5.00
21	4.50	5.00	4.75	4.00	4.25	3.50	5.00
Mean	2.68	2.44	2.14	1.73	2.10	2.20	3.13

There was no significant color change in day 1–5, whereas textural quality remained the same from day 1–3 (Table 3) . However, by day 11 the fruits were shown to have a significant color change (*p* > .05), but it did not differ significantly from days 8 to10. Results indicate that as the days progressed, color, and textural quality of tomatoes under refrigeration depreciated, however, quality parameters of color and texture remained acceptable for a period of 11 days in cold storage.

**Table 3:** The mean of the effect of days on color and texture in cold storage conditions

Day	Color (cold)	Texture (cold)
1	1.0000c	1.0000d
2	1.0000c	1.0000d
3	1.0000c	1.0000d
4	1.0000c	1.3214d
5	1.0000c	13214d
6	1.8214b	1.7500c
7	1.9286b	1.8929bc
8	2.1429ab	1.9286bc
9	2.1786ab	2.2143ab
10	2.1786ab	2.2500ab
11	2.3571a	2.3571a
LSD	0.4063	0.4088

LSD, least significant difference.

Table 4 shows the treatment (ppm) mean on the color and texture of tomatoes under storage. However, concentration 15 ppm (cold) was recorded to have the worst color change, whereas 7.5 ppm had the lowest mean on color change which indicated that the color assessment of tomatoes quality was acceptable and 15.0 ppm had the highest mean on texture change. Ranking results indicate that tomatoes treated with 15 ppm of potassium permanganate were not of acceptable quality in terms of color and texture.

**Table 4:** The treatment mean of color and texture under cold storage conditions

Treatment (ppm)	Mean color (Cold)	Mean textures (Cold)
0	1.7727b	1.8636b
2.5	1.6591bc	1.5909bcd
5	1.5000bc	1.5227bcd
7.5	1.0909d	1.0682e
10	1.4318c	1.3636de
12.5	1.7273bc	1.7727bc
15	2.1818a	2.2955a
LSD	0.3241	0.3261

LSD, least significant difference.

The best quality tomatoes were with treatment concentration of 7.5 ppm. However, the 7.5 ppm did not differ statistically (*p* > .05) from concentration 10.0 ppm (Table 5).

**Table 5:** The mean effect of KMnO_4_ on the color and texture of tomato under cold storage conditions

Treatment (ppm)	Mean color	Mean texture
0	2.44	2.68
2.5	2.29	2.44
5	2.10	2.14
7.5	1.64	1.73
10	2.10	2.10
12.5	2.15	2.20
15	2.80	3.13

## 4 Discussion

The study has shown that both the treated and untreated tomatoes went through the different stages of ripening. It also showed that potassium permanganate has a preservative effect on tomatoes, although tomatoes under cold storage conditions were known to perform better. This concurs with Sudha, Amutha, Muthulaksmi, Baby Rain, and Mareeswani (2007) that certain chemicals are known to delay ripening to reduce losses and to improve and maintain the color and quality by slowing down the metabolic activities of the fruit. These chemicals are reported to arrest the growth and spread of microorganism by reducing the shriveling which ultimately leads to an increase in shelf life (Sudha et al., 2007). The texture played an important role in acceptability of the tomato fruits. Texture is an important attribute to evaluate the quality of tomato fruit and it is determined by the fruit morphological and physiological characteristics: epicarp firmness and maturity stage (Sharma, Yamdagni, Gaur, & Shukla, 1996). This study, however, showed that the texture score increases with the passage of ripening stages in all fruits. However, in cold storage conditions ripening was delayed longer than the tomato in ambient conditions due to lower temperature. This concurs with Bourne (1983) that low temperature is used to preserve tomato by lowering microbial activity through the reduction in microbial enzymes which in turn leads to an increased shelf life.

Other quality parameters not identified in this research include weight loss and loss in flavor which could be attributed to potassium permanganate activities in delaying ripening. Potassium permanganate is said to be an ethylene degrading chemical which degrades ethylene into water and carbon dioxide. According to Roth (1999) water observed in jars created a high humid environment retarding transpiration and water loss in ethylene preserved fruits. Observation at eye view showed that control displayed rapid increase in weight loss under ambient conditions than other treatments. This strongly aggress with Kader (1994) that tomato being a climacteric and perishable vegetable has a very short life span. The level of delayed ripening confirms that potassium permanganate (KMnO_4_) is effective in delaying ripening in tomato, maintaining tissue firmness in the fruit, and also reduce respiration which is in line with Chaplin and Scott (1980) that KMnO_4_ not only delay the ripening process but also maintains its firmness and vigor.

## Funding Information

No funding information provided.

## Conflict of Interest

None declared.
